# Congenital Stationary Night Blindness: Structure, Function and Genotype–Phenotype Correlations in a Cohort of 122 Patients

**DOI:** 10.1016/j.oret.2024.03.017

**Published:** 2024-09

**Authors:** Mohamed Katta, Thales A.C. de Guimaraes, Yu Fujinami-Yokokawa, Kaoru Fujinami, Michalis Georgiou, Omar A. Mahroo, Andrew R. Webster, Michel Michaelides

**Affiliations:** 1 UCL Institute of Ophthalmology, University College London, London, United Kingdom; 2 Genetics Department, Moorfields Eye Hospital, London, United Kingdom; 3 Laboratory of Visual Physiology, Division of Vision Research, National Institute of Sensory Organs, NHO Tokyo Medical Center, Tokyo, Japan; 4 Department of Health Policy and Management, Keio University School of Medicine, Tokyo, Japan; 5 Jones Eye Institute, University of Arkansas for Medical Sciences, Little Rock, Arkansas

**Keywords:** Congenital stationary night blindness, Inherited retinal disease, Natural history, Genotype, Phenotype

## Abstract

**Objective:**

To examine the molecular causes of Schubert–Bornschein (S–B) congenital stationary night blindness (CSNB), clinically characterize in detail, and assess genotype–phenotype correlations for retinal function and structure.

**Design:**

Retrospective, longitudinal, single-center case series.

**Participants:**

One hundred twenty-two patients with S–B CSNB attending Moorfields Eye Hospital, United Kingdom.

**Methods:**

All case notes, results of molecular genetic testing, and OCT were reviewed.

**Main Outcome Measures:**

Molecular genetics, presenting complaints, rates of nystagmus, nyctalopia, photophobia, strabismus, color vision defects and spherical equivalent refraction (SER). Retinal thickness, outer nuclear layer (ONL) thickness, and ganglion cell layer + inner plexiform layer (GCL+IPL) thickness from OCT imaging.

**Results:**

X-linked (*CACNA1F* and *NYX*) and autosomal recessive (*TRPM1, GRM6, GPR179* and *CABP4*) genotypes were identified. The mean (± standard deviation) reported age of onset was 4.94 ± 8.99 years. Over the follow-up period, 95.9% of patients reported reduced visual acuity (VA), half had nystagmus, and 64.7% reported nyctalopia. Incomplete CSNB (iCSNB) patients more frequently had nystagmus and photophobia. Nyctalopia was similar for iCSNB and complete CSNB (cCSNB). Color vision data were limited but more defects were found in iCSNB. None of these clinical differences met statistical significance. There was no significant difference between groups in VA, with a mean of 0.46 logarithm of the minimum angle of resolution, and VA remained stable over the course of follow-up. Complete congenital stationary night blindness patients, specifically those with *NYX* and *TRPM1* variants, were more myopic. *CACNA1F* patients showed the largest refractive variability, and the *CABP4* patient was hyperopic. No significant differences were found in OCT structural analysis during the follow-up period.

**Conclusions:**

Retinal structure in CSNB is stationary and no specific genotype–structure correlates were identified. Visual acuity seems to be relatively stable, with rare instances of progression.

**Financial Disclosure(s):**

Proprietary or commercial disclosure may be found in the Footnotes and Disclosures at the end of this article.

Congenital stationary night blindness can be classified into 2 types based on electroretinographic findings^1^: (i) the Riggs type, which is much less prevalent, where there are reduced a- and b-waves in response to a scotopic bright flash; and (ii) the Schubert–Bornschein (S–B) type with selective loss of the b-wave.^2^, ^3^, ^4^ The Riggs type of congenital stationary night blindness (CSNB) has been associated with variants in *GNAT1*, *PDE6B*, *RHO*, and *SCL24A1*.^5^, ^6^, ^7^, ^8^ The Schubert–Bornschein type of CSNB is characterized by signal transmission dysfunction between photoreceptors and bipolar cells.^9^ Based on the difference in dark adapted electroretinography (ERG) findings, patients were classified as incomplete CSNB (iCSNB) with residual rod system function, as assessed with the dim-flash ERG, or complete CSNB (cCSNB), with no detectable dim-flash ERG. Dysfunction of either the ON bipolar cells alone for cCSNB or both the ON and OFF bipolar cells, because of the abnormal glutamate release from the photoreceptors in iCSNB, has been demonstrated electrophysiologically.^10^^,^^11^ Since the advent of molecular genetic testing, 6 genes have been found to cause this S–B type of CSNB. Two genes are responsible for iCSNB: *CACNA1F*^12^ and *CABP4*^13^; and 4 genes are associated with cCSNB: *NYX,*^14^^,^^15^ *TRPM1,*^16^, ^17^, ^18^ *GRM6,*^19^ and *GPR179.*^20^ It has been noted that night blindness is not consistently a feature in iCSNB, particularly in *CABP4*-associated disease, and, hence, the term cone–rod synaptic disorder has been proposed for the latter condition.^21^, ^22^, ^23^, ^24^

The advancements in molecular biology, retinal imaging, and genetics led to the first commercially available gene therapy for an inherited retinal disease,^25^ with many clinical trials underway.^26^ Congenital stationary night blindness could pose an attractive candidate for gene therapy given its “stationary” nature and gross retinal structural integrity,^8^ but natural history studies for the disease are limited.

We present the first large longitudinal study assessing genotype–phenotype correlations of both function and structure in S–B CSNB.

## Methods

This retrospective study adhered to the tenets of the Declaration of Helsinki and received approval from the Moorfields Eye Hospital ethics committee, and the requirement for informed consent was waived. Patients with a confirmed molecular diagnosis of CSNB were identified from the genetics database at a single tertiary referral center (Moorfields Eye Hospital, London, UK). In the study, we investigated patients aged <50 years at presentation, with S–B CSNB phenotype and molecular confirmation. For the longitudinal analyses ≥2 visits, ≥1 year apart, were required. Exclusion criteria were any ocular comorbidity or cataract or refractive surgery. All patients were seen by retina specialists.

### Data Collection

Clinical and electronic patient notes were examined and the presenting history along with clinical assessments including best-corrected visual acuity (BCVA), color vision assessed with Ishihara plates, refraction, slit-lamp biomicroscopy, and fundoscopy findings were extracted. Best-corrected visual acuity was measured monocularly in either Snellen or logarithm of the minimum angle of resolution (logMAR) notation, with Snellen acuities converted to logMAR. Subjective or objective refraction was undertaken by an optometrist for both adults and children, and the spherical equivalent refraction (SER) was used for analysis.

### OCT Imaging

Volumetric spectral-domain OCT (SD-OCT, Spectralis; Heidelberg Engineering Ltd) scans were analyzed for structural retinal changes using the HEYEX2 software by 2 experienced graders (M.K. and T.A.C.G.). Images were automatically segmented for the different retinal layers and further adjusted manually if required. The 1-, 3-, 6-mm ETDRS circle was manually centered over the anatomic fovea and retinal thickness taken from all 4 quadrants of the 3-mm circle and the central 1-mm circle. Retinal thickness was determined using the built-in software by measuring the distance between the retinal pigment epithelium and inner limiting membrane borders. Ganglion cell layer plus inner plexiform layer (GCL+IPL) thickness and foveal outer nuclear layer (ONL) thickness were calculated, as previously described and illustrated in Figure S1 (available at www.ophthalmologyretina.org).^27^^,^^28^ Patients with foveal hypoplasia or posterior staphyloma were noted.

### Genetic Analysis

Genomic DNA was isolated from peripheral blood lymphocytes (Gentra Puregene Blood Extraction Kit; Qiagen). A combination of Sanger sequencing and next-generation sequencing, including a panel of retinal dystrophy genes, whole exome sequencing, and whole genome sequencing, was used to identify variants in 6 causative genes for S–B CSNB: iCSNB (*CACNA1F*, *CABP4*), cCSNB (*NYX, TRPM1, GRM6, GPR179*). All recruited patients were reassessed for their detected variants, as described in Methods S1 (available at www.ophthalmologyretina.org).

### Statistical Analysis

IBM SPSS statistics v.29.0 was used for all statistical analyses. Categorical data were analyzed with chi-square tests. Mixed-effects models were used to analyse BCVA and SER. Analyses were run with BCVA or SER as the dependent variable, with genotype or S–B subtype and age as fixed factors. Sex was added as a fixed factor along with age for the analyses of *TRPM1* and *GRM6* genotypes. The subjects were set as the random factor. Random intercept and slope were incorporated into the models. An intraclass correlation coefficient was calculated for each analysis. An intraclass correlation coefficient value closer to 1 suggests that greater intrasubject variability over time in the dependent variable, visual acuity (VA) or SER, explains the total variance of the model. OCT data variables were checked for normality and homogeneity of variance and analyzed with parametric methods (*t* test, univariate and multivariate analyses). Statistical significance was set at *P* < 0.05. Bonferroni adjustments of *P* values were conducted for multiple comparisons and are indicated where applied.

## Results

### Demographic and Genetics

We identified 122 eligible patients (male: n = 101 [83%]) from 107 pedigrees. The mean age at first examination was 8.66 (range, 0.7–50; standard deviation [SD], 10.29) years, and the mean follow-up time was 7.81 (range, 1–47; SD, 8.16) years. There were 59 patients (48.4%) with the *CACNA1F* genotype, 25 with *NYX* (20.5%), 21 with *TRPM1* (17.2%), 14 with *GRM6* (11.5%), 2 with *GPR179* (1.6%) and 1 with *CABP4* patient (0.8%). The sex, age at baseline, and last follow-up by genotype are presented in Table S1 (available at www.ophthalmologyretina.org). Table S2 (available at www.ophthalmologyretina.org) provides further demographics by analysis type.

### Symptoms and Examination Findings

The age of onset of symptoms was available in 96 of the patients, with mean ± SD age at onset of 4.94 ± 8.99 years. Table 3 shows the frequency of presenting complaints in the cohort and their prevalence by the end of follow-up. The most prevalent presenting complaints were reduced BCVA (55.7%), nystagmus (38.5%), nyctalopia (29.5%), and myopia (22.9%), with squint (15.5%) and photophobia (1.6%) being less frequent. One patient was asymptomatic at the time of diagnosis and was only diagnosed after genetic testing due to an affected sibling. The most common combined feature was reduced VA with myopia that did not improve with correction (28.6%), raising suspicion of underlying retinal pathology from the referring hospital or optometrist.

**Table 3:** Presenting Complaints and Features during Follow-Up

	At Presentation		By End of Follow-Up		Age of onset (yrs), mean ± SD
	Number	%	Number	%	
Reduced BCVA	68	55.74	117	95.9	4.11 ± 5.83
Nystagmus	47	38.52	63	51.64	1.91 ± 4.13
Nyctalopia	36	29.5	79	64.75	8.71 ± 10.14
Myopia	28	22.95			
Squint	19	15.58			
Photophobia	2	1.64	8	6.56	14.60 ± 15.43
Known CSNB in family	1	0.82			
Reduced BCVA and myopia	35	28.69			
Reduced BCVA, nyctalopia, nystagmus	6	4.92			
Reduced BCVA, myopia, nystagmus	6	4.92			

BCVA = best-corrected visual acuity; CNSB = congenital stationary night blindness; SD = standard deviation.

By the end of the follow-up period, the majority of patients (95.9%) reported reduced BCVA. Half of the patients had nystagmus. The number of patients reporting nyctalopia increased to 64.7% of the cohort and there were more patients reporting photophobia (6.5%). The patient reports of nyctalopia and photophobia appeared at an older mean ± SD age, (8.71 ± 10.14 and 14.60 ± 15.43 years) compared with other symptoms. Table 4 shows the clinical features found by S–B subtype and genotype. Similar proportions of patients with iCSNB (63.3%) vs. cCSNB (66.1%) reported nyctalopia (chi-square, 0.104, 1 degree of freedom [df], n = 122; *P* = 0.747). There were a greater number of patients with nystagmus in the iCSNB subgroup (60%) vs. cCSNB (43.5%), but this difference did not meet statistical significance (chi-square, 3.305 [1df, n = 122], *P* = 0.069). Similarly, more iCSNB patients reported photophobia (7 vs. 3 patients) than the cCSNB subgroup; again, not meeting statistical significance (chi-square, 2.284 [1 df, n = 122], *P* = 0.131). There was no significant difference in strabismus between the 2 groups. Color vision data were only available for 18 patients with iCSNB and 11 with cCSNB. There were a greater number with color defects in iCSNB (33.3% vs. 18.1%), but this was not significant (chi-square, 0.79 [1df, n = 29], *P* = 0.376).

**Table 4:** Clinical Features by S-B CSNB Subtype and Genotype

Clinical feature	All cCSNB		*NYX*		*TRPM1*		*GRM6*		*GPR179*	
	%	n/N	%	n/N	%	n/N	%	n/N	%	n/N
Nystagmus	43.55	27/62	44	11/25	33.33	7/21	35.71	5/14	50	1/2
Nyctalopia	66.13	41/62	64	16/25	71.43	15/21	28.57	4/14	0	0/2
Photophobia	3.23	2/62	4	1/25	0	0/21	7.14	1/14	0	0/2
Strabismus	32.26	20/62	40	10/25	33.33	7/21	14.29	2/14	50	1/2
Color defect	18.18	2/11	28.57	2/7	0	0/3	0	0/1	0	0/0

Clinical feature	All iCSNB		*CACNA1F*		*CABP4*	
	%	n/N	%	n/N	%	n/N
Nystagmus	60	36/60	59.32	35/59	100	1/1
Nyctalopia	63.33	38/60	64.41	38/59	0	0/1
Photophobia	10	6/60	10.17	6/59	0	0/1
Strabismus	20	12/60	20.34	12/59	0	0/1
Color defect	33.33	6/18	33.33	6/18	0	0/0

cCSNB = complete congenital stationary night blindness; iCSNB = incomplete congenital stationary night blindness; n/N = number/total number of patients; S–B = Schubert–Bornschein.

**Figure 2:**
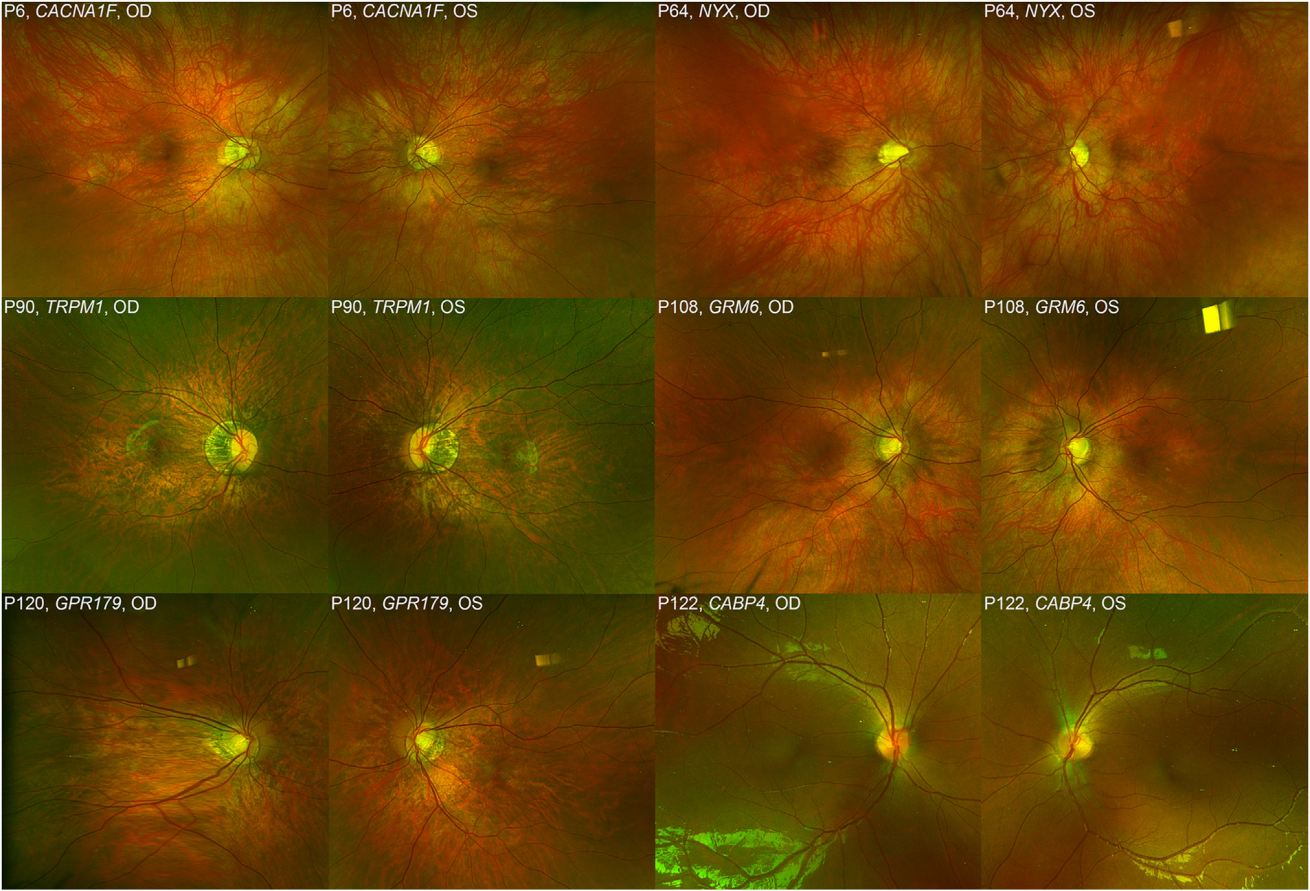
Examples of fundus photographs of both eyes in all 6 genotypes. Typical features displayed of tilted optic discs, peripapillary atrophy, and pronounced choroidal vasculature in both eyes giving the fundi a tessellated appearance. The patient with *CABP4* genotype does not show these myopic features as they are hyperopic. OD = right eye; OS = left eye.

Myopic posterior pole changes of tilted discs, peripapillary atrophy, and a tessellated fundus were the most commonly noted fundoscopic features in 48 patients (39.3%), with a normal examination in the rest of the patients. Posterior staphyloma was noted on clinical examination in 3 patients (2.4%). Figure 2 shows the typical fundoscopic features in all 6 genotypes.

### Visual Acuity

Mean VA of all visits was slightly better in right eyes than left eyes (0.46 ± 0.24 vs. 0.49 ± 0.27 logMAR; *t*(554 df) = −3.12; *P* = 0.002). Because the difference was not clinically relevant and for the purposes of brevity, the analysis presented here is for right eyes only. Based on the United Kingdom’s Driver and Vehicle Licensing Agency VA cutoff of better than 6/12 Snellen acuity with both eyes open, 63 patients (51.6%) would be expected to be legal to drive subject to meeting all other requirements. Only 4 patients lost ≥0.2 logMAR VA over the course of follow-up. Details of the course of those 4 patients are provided in Results S1 (www.ophthalmologyretina.org).

Table S5 (available at www.ophthalmologyretina.org) shows the results of a mixed-effects model examining VA extrapolated to BCVA at birth by genotype. There was no statistically significant difference in predicted VA at birth in comparison to that of the *CACNA1F* patients (0.56 logMAR) in the genotype analysis or between complete vs. incomplete in the S–B subtype analysis. Pairwise comparisons of the mean BCVA between genotypes and S–B subtype with age as a covariate and Bonferroni adjustments for multiple comparisons showed no statistically significant difference between the groups. The *NYX* patients showed a statistically significant improvement in VA in comparison to *CACNA1F* patients, but the effect size was small, 0.006 logMAR, equivalent to approximately 0.3 letters per year. There was no significant difference in pairwise analysis of mean VA between males and females, with age as a covariate, in *TRPM1* (0.39 vs. 0.45 logMAR, respectively; *P* = 0.52) or *GRM6* patients (0.31 vs. 0.27 logMAR, respectively; *P* = 0.24).

### Refractive Error

There was no significant difference in mean SER between right and left eyes (−6.64 ± 4.82 vs. −6.62 ± 4.81 diopters (D); *t*(426 df) = −0.33; *P* = 0.74). Right eye data were used for the analysis presented here. Table S6 (available at www.ophthalmologyretina.org) displays the results of the mixed-effect model examining SER by genotype using 427 patient visits and S–B subtype, which include the 2 *GPR179* and 1 *CABP4* patients using 455 visits, extrapolated to SER at birth. *TRPM1* was further analyzed with sex as an additional fixed factor with 80 patient visits. *NYX* and *TRPM1* patients are predicted to be significantly more myopic at birth in comparison to *CACNA1F* patients (−6.57 and −6.19 vs. −3.42 D, respectively, *P* < .05), with cCSNB subtype patients significantly more myopic than iCSNB patients (−5.92 vs. −3.24 D, respectively, *P* < 0.01). Only *NYX* patients showed a significant increased rate of progression of myopia (−0.192 D per year, *P* < 0.05) in comparison to *CACNA1F* patients. There was a trend to an increased rate of myopia progression in the cCSNB subgroup, but this did not meet statistical significance (*P* = 0.071). Pairwise analysis comparing mean SER between the genotypes, with age as a covariate and Bonferroni adjustments for multiple comparisons, only showed significant differences between *CACNA1F* and *NYX* (−4.58 vs. −8.47 D, *P* = 0.009) and between *CACNA1F* and *TRPM1* patients (−4.58 vs. −7.81 D, *P* = 0.046). Similarly, pairwise analysis of mean SER between complete and incomplete subtypes, controlling for age, (−7.50 vs. −4.38 D, respectively; *P* = 0.001), showed a more myopic mean for the complete subtype. Adding gender as a fixed factor to the *TRPM1* model did not find a statistically significant difference between males and females, despite females predicted to be 1.53 D more myopic than males at birth, with almost twice the rate of progression of males. This is likely because of the smaller number of patients in the model’s subgroups. Similarly, there was a trend toward increased myopia progression in females not meeting statistical significance (*P* = 0.085). Furthermore, comparison of mean SER between male and female patients with *TRPM1,* with age as a covariate, found that females were more myopic than males (−9.59 vs. −6.93 D, respectively; *P* = 0.18) but again not meeting statistical significance. A mixed-effects model analysis was not conducted for *GRM6* patients, but univariate analysis controlling for age showed no statistical difference between mean SER for males and females (−5.56 vs. −4.19 D, respectively; *P* = 0.55). By the end of follow-up there were only 4 hyperopic patients, 3 *CACNA1F* patients with mean ± SD SER +1.23 ± 0.97 D and the single *CABP4* patient, SER +2.63 D.

### OCT Structural Analysis

**Figure 3:**
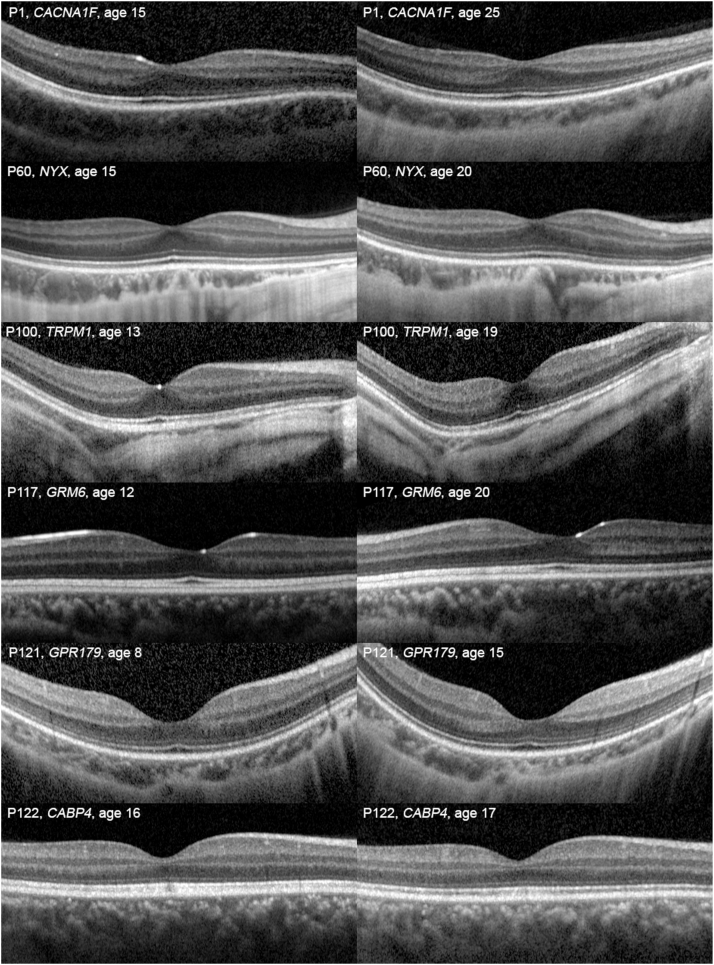
Longitudinal OCT imaging in congenital stationary night blindness. All genotypes displayed stable retinal structure as assessed by OCT imaging over the follow-up period.

A paired *t* test between right and left eye data showed no significant difference between the eyes in any of the OCT variables collected and so only right eye data are presented here. A single OCT imaging session was available for 75 patients, and longitudinal OCT data were available for 51 patients, with a mean ±SD follow-up of 4.56 ± 2.97 years. Table S7 (available at www.ophthalmologyretina.org) displays the means of the OCT measures and results of a paired *t* test which showed no significant differences between the first and last visit means. Table S8 (available at www.ophthalmologyretina.org) shows the results of a multivariate analysis of the OCT measures between the S–B subtypes controlling for age and SER at the patient’s last visit. This largely showed no significant differences between the incomplete and complete subtypes, apart from slightly increased superior parafoveal retinal thickness and nasal GCL+IPL thickness in the complete subtypes. Figure 3 shows examples of longitudinal OCT imaging in patients with CSNB. There was no ellipsoid zone disruption in any of the patients over the course of follow-up.

Foveal hypoplasia was present in 17 patients (22.6%) and posterior staphyloma in 6 out of 75 patients (8%). Univariate analyses with age as a covariate revealed a trend to reduced BCVA in patients with foveal hypoplasia (mean difference, −0.11 logMAR; F, 2.390; *P* = 0.126) and, to a lesser degree, posterior staphyloma (mean difference, −0.09 logMAR; F, 0.814; *P* = 0.37), in comparison to patients without these features, but both of these analyses did not meet statistical significance. There were more iCSNB patients with foveal hypoplasia, 11 patients (28.2%), compared with cCSNB 6 patients (16.6%), but no statistically significant difference was observed (chi-square [1 df, n = 75] = 1.422, *P* = 0.233).

### Genetic Analyses

In total, 105 variants were identified in 6 causative genes in 122 patients (Table S9, available at www.ophthalmologyretina.org). Forty-two *CACNA1F* variants in 59 patients from 50 families, 19 *NYX* variants in 24 patients from 22 families, 27 *TRPM1* variants in 22 patients from 19 families, 14 *GRM6* variants in 14 patients from 13 families, 2 *GPR179* variants in 2 patients from 2 families, and 1 *CABP4* variant in 1 patient were detected. There were 52 novel variants (52/105, 49.5%) that have never been reported in clinical cases.

The detailed molecular genetic analyses results are presented in Table S10 (available at www.ophthalmologyretina.org). Various types of variants were identified, including 45 missense (45/105, 42.8%), 21 frameshift (21/105, 20.0%), 17 splice site alteration (17/105, 16.2%), and 14 stop (14/105, 13.3%) variants. A higher proportion of null variants suggesting loss of function was detected in *CACNA1F* (31/42, 73.8%), whereas a higher proportion of non-null variants was found in *NYX* (15/19, 78.9%). There were 30 pathogenic variants, 38 likely pathogenic variants, and 37 variants of uncertain significance according to the American College of Medical Genetics and Genomics guidelines. A higher prevalence of pathogenic/likely pathogenic variants was observed in *CABP4* (1/1, 100.0%), *CACNA1F* (33/42, 78.5%), and *GRM6* (9/14, 64.3%).

### Intravariant vs. Intragenotype Phenotypic Variability

The variances in BCVA and SER at the last visit were compared for 3 groups with *CACNA1F* variants and similarly for *NYX* variants. The first *CACNA1F* group included 5 patients from 2 families with the c.3052G>A variant. The second *CACNA1F* group included 4 patients from 3 families with the c.3269+1G>A variant, with the final group being all other *CACNA1F* variants in the cohort. The first *NYX* group consisted of 3 patients from 2 families with the c.647A>G variant, the second group with 3 patients from 2 families with the c.411_419dup variant, and the third group being all other *NYX* variants. Using nonparametric Levene test of variance, there was no significant difference in the variance between members of the same variant compared with their respective genotype cohort for BCVA (*CACNA1F* [*P* = 0.083] and *NYX* [*P* = 0.165]) or SER (*CACNA1F* [*P* = 0.252] and *NYX* [*P* = 0.451]).

## Discussion

Herein, we analyzed 122 patients with S–B CSNB, including the main 6 genotypes: *CACNA1F* and *CABP4* for iCSNB and *NYX*, *TRPM1*, *GRM6*, and *GPR179* for cCSNB. No phenotype–genotype association was observed among the different genotypes, but a distinction between iCSNB and cCSNB was apparent.

The distinction between iCSNB and cCSNB has been attributed to differing impacts on the ON and OFF bipolar cell pathways.^8^^,^^29^ Genes involved in iCSNB encode proteins at the presynaptic terminals, leading to dysfunction of both ON and OFF pathways; whereas, in cCSNB, encoded proteins are in the postsynaptic ON bipolar dendrite, limiting dysfunction to the ON pathway.^8^ Whereas cones synapse directly with ON and OFF bipolar cells, rods synapse directly only with ON bipolar cells. It is therefore plausible that iCSNB will display a greater degree of cone pathway dysfunction, with worse VA, color vision, and photophobia, than cCSNB, where the rod pathway impairment may be greater. Indeed, although not statistically significant, more of our cCSNB patients had nyctalopia, and fewer had photophobia in comparison to the iCSNB patients. In large cohorts, nyctalopia has been reported in 50% to 60% of iCSNB patients^29^, ^30^, ^31^ and 60% to 100% of cCSNB patients;^29^^,^^32^, ^33^, ^34^ these figures are in keeping with those in our study (63.3% and 66.1%, respectively). The lack of a significant difference between the 2 groups may be due to the subjective nature of nyctalopia. Bijveld et al^35^ studied the nyctalopia of both subtypes with a questionnaire and dark adapted visual function tests and found that although most cCSNB patients experience nyctalopia, most did not describe these as severe and their visual field was normal once light levels exceeded a relatively sharp threshold, around the level of moonlight. This may explain the similar reported rates between iCSNB and cCSNB in our study.

We also report lower rates of photophobia in our cohort (10% iCSNB vs. 3.2% cCSNB) than other groups, who have reported 44% to 53% in iCSNB^29^^,^^31^ and 21% in cCSNB.^29^ This may be due to photophobia being a subjective trait of CSNB depending on the lighting environment and the ability of the patient to convey this more nuanced symptom, which may also be more challenging to describe at younger ages. We found that the mean age of photophobia reports was older, at 14.6 years, compared with 8.7 years for nyctalopia.

Color vision was only documented for 29 of our patients. We found a greater number of color defects in the iCSNB subgroup compared with cCSNB (33.3% vs. 18.1%). This was similar to Bijveld et al^29^ who found 47% of their iCSNB cohort had mild-severe defects and 14% of their cCSNB cohort had only mild defects. Reported prevalence of nystagmus varies widely, with 35% to 66% in iCSNB^9^^,^^29^^,^^31^^,^^36^ and 20% to 78% in cCSNB^9^^,^^29^^,^^32^^,^^33^; our cohort prevalences fall within these ranges. Pieh et al^37^ studied the nystagmus of a small CSNB cohort (n = 10) and found all had pendular high-frequency, low-amplitude nystagmus regardless of S–B subtype. In our cohort, nystagmus type was rarely recorded, but, in all 6 patients for which it was recorded, it was pendular. Strabismus is another feature that varies significantly between reports, with 10% to 100% prevalence in iCSNB and 17% to 86% in cCSNB, with most studies apart from Allen et al^38^ reporting greater prevalence in cCSNB.^9^^,^^29^^,^^30^^,^^32^^,^^38^ Similarly, we found more strabismus in cCSNB (32.3%) compared with iCSNB (20%). The prevalence of strabismus was not associated with VA.

Fundus examination revealed either no pathological features or features consistent with high myopia (Figure 2).^9^^,^^29^^,^^30^ Contrary to the classically taught triad of CSNB features of reduced VA, nyctalopia and nystagmus, most patients presented with either reduced vision or reduced vision and myopia. Nyctalopia is a trait that may not be readily reported in younger ages, which has been suggested by other groups.^33^^,^^39^ It is important to consider a diagnosis of CSNB or other inherited retinal disease early on in children who present with reduced vision and myopia, or with BCVA not correcting with glasses, so they may access the necessary diagnostic services and support promptly.

Examining this large cohort, with 555 visits over a mean follow-up of 8.3 years, revealed stable BCVA over time. The mean VA was 0.46 logMAR and using mixed-effects models we found no clinically significant change over time, no statistical difference between the S–B subtypes or genotypes or between sexes in autosomal recessive *TRPM1* and *GRM6* patients. The lack of BCVA difference between the 2 subtypes has been previously reported.^9^^,^^38^^,^^40^ This contrasts with the worse VA that has been found in iCSNB compared with cCSNB in other studies.^29^^,^^32^^,^^36^^,^^41^^,^^42^ Similar to the present study, Bijveld et al^29^ found no statistical difference in VA within the cCSNB subtypes. We predicted from our data that over half of our patients would meet BCVA standards required for a driving license in the United Kingdom (UK). Similarly, Bijveld et al,^35^ in another study, found 36.3% of their 22 patients held a driver’s license with or without conditions. Only 4 patients out of our 110 who were followed up lost ≥2 lines of logMAR VA. Only 2 of these experienced >2 lines of worsening and both had *CACNA1F* variants. Similarly, there have been other rare reports of visual deterioration, almost all in iCSNB genotypes.^36^^,^^43^, ^44^, ^45^, ^46^ Hove et al^36^ found progressive VA loss in 2 out of 60 patients (3.3%) with iCSNB,^36^ very similar to our rate (3.6%). Patients with *CABP4* variants are rare, but those reported tend to have worse than average VA, ranging from 0.7 to 1.3 logMAR.^13^ Our single *CABP4* patient had a BCVA of 0.79 logMAR at last visit.

We describe the refractive error of the different genotypes over time using a mixed-effects model to take into account 427 objective/subjective refractions. *NYX* and *TRPM1* patients were predicted to be significantly more myopic at birth (−6.57 and −6.19 D, respectively), and *NYX* patients to progress faster (−0.19 D/year) compared with *CACNA1F* patients (−3.4 D progressing at −0.11 D/year). Incomplete congenital stationary night blindness patients were found to be on average >3 D less myopic than cCSNB patients (−4.38 vs. −7.5 D); however, there was no significant difference found in myopia progression between the 2 groups. There is increasing evidence that high myopia can be induced with ON bipolar-pathway dysfunction,^39^ although there is also evidence implicating the OFF pathway.^47^ Hendriks et al^48^ found that *NYX* and *TRPM1* patients were in the top 3 myopic groups in a cross-sectional study in 302 patients with inherited retinal diseases. They also found *CABP4* to be the genotype with the highest hyperopia.^48^ Indeed, nearly all reports of *CABP4* have been hyperopic^13^^,^^24^^,^^49^ including our single patient with +2.63 D SER. *CACNA1F* patients had the highest variability in refractive status, with 3 out of the 4 hyperopic patients by the end of follow-up having variants in this gene. Similarly, other groups have found the *CACNA1F* genotype to have significant rates of hyperopia, up to 22% in Bijveld et al.^9^^,^^29^^,^^30^^,^^41^^,^^50^

To examine whether certain variants caused a certain VA and refraction compared with other variants within the same gene, we compared 3 groups from the *CACNA1F* and *NYX* sub-groups. Two groups from each genotype contained patients with the 2 most common variants, with the third group consisting of all the other variants in that subgroup. There was no difference found in the variance in BCVA or SER between these 3 groups in either the *CACNA1F* or *NYX* cohorts. This agrees with other reports finding that clinical phenotype was not variant specific in *CACNA1F*^29^^,^^30^^,^^35^^,^^50^ or *NYX*.^34^

Functional stability of CSNB patients over time has been reported in this study and others, but longitudinal retinal structural analysis in a large cohort has not been reported previously. We found no significant change in retinal thickness, ONL thickness, or GCL+IPL thickness over a mean follow-up period of 4.5 years. Controlling for age and SER between S–B sub-groups, we found only slightly increased superior parafoveal retinal thickness and nasal GCL+IPL thickness nasally in the cCSNB subgroup. In a study of 3 *GRM6* patients (not included in the current cohort) compared with 93 normal controls, Godara et al^51^ found reduced GCL+IPL thickness with a normal ONL thickness. However, it is not clear whether the controls were myopic. If the controls were emmetropes this could lead to apparent thinning in the CSNB patients who will have been myopic. The mean GCL+IPL thickness they found in 3 patients was similar to our cohort, 62.67 μm and 69.37 μm, respectively.^51^ Chen et al^52^ studied 5 patients with iCSNB and compared them to 4 myopic but otherwise healthy controls and found the parafoveal GCL+IPL, inner nuclear layer, total receptor, and total retinal thickness to be reduced in the iCSNB patients. The measurements of the oldest patient, aged 69 years, fell within that of the younger patients, leading the authors to conclude that the changes are stationary,^52^ which agrees with the stability in retinal structure found in the present study. In a qualitative study, Al Oreany et al^53^ found the total retinal thickness of 2 CSNB twins did not differ from 5 myopic, age matched controls, but the inner nuclear layer appeared thinned. Recently, Parodi et al^54^ studied 3 patients (2 *TRPM1*, 1 *CACNA1F*) compared with myopic healthy controls and found normal central macula thickness, reduced choroidal thickness, and increased outer plexiform layer thickness with ONL thinning in the CSNB patients. Foveal pit volume was found to be reduced in a study of 22 iCSNB patients in comparison to 64 normal controls^36^; again, these results may have been seen by comparison to an emmetropic patient cohort where the retinal thickness and, therefore, foveal volume is expected to be greater than a myopic cohort. These results, taken together, may suggest a thinning of the inner retinal layers while relative sparing of the outer retinal layers; however, a large cross-sectional study with matched myopic controls is required to answer this conclusively. Foveal hypoplasia has been reported at 33% to 58% in other studies, mainly in iCSNB.^36^^,^^54^ We also found foveal hypoplasia more commonly in iCSNB (28.2%) compared with cCSNB (16.6%), with no statistical difference, likely due to the small number of patients. There was no statistical association between the presence of foveal hypoplasia or posterior staphyloma and worse VA, suggesting a disconnect between structure and function in CSNB, as also noted in many other inherited retinal disorders.

One hundred five variants in 6 genes were identified in 122 patients, including 52 novel variants. Variable types of variants detected for cCSNB and iCSNB suggested an advantage of comprehensive high-throughput sequencing methods to detect disease-causing variants in S–B CSNB. The high proportion of novel variants for each causative gene highlights the importance of pathogenicity assessment, given the high prevalence of missense variants. For some genes (*NYX, TRPM1*), several transcript versions have been applied previously and different descriptions for each variant have been found; thus, accurate variant assessment based on particular transcript ID helps to interpret all variants comprehensively.

Retrospective studies such as this are useful in studying rare diseases such as CSNB but inevitably have limitations. Because the data have not been collected in a standardized research study pro forma, there were missing data and some data, particularly symptomology, are prone to recall bias. Electroretinogram findings were not presented here because these have already been reported extensively.^8^

This study has found a stable natural history in terms of visual function and retinal structure in CSNB, with over half of patients expected to have VA good enough to drive in the daytime. Further studies on CSNB night vision dysfunction are needed to assess baseline mobility at different light levels. This, together with further quality of life studies, may inform us of patients’ views on possible interventional therapies that may not be popular given the apparent high level of functioning in this inherited retinal disease compared with others currently under clinical trial. Structural studies compared with a matched myopic cohort are also needed to conclusively answer questions relating to structural integrity in CSNB, which will be important for consideration of CSNB as a candidate disease for intervention.

In conclusion, iCSNB displays more cone-related dysfunction such as color vision defects and photophobia, likely stemming from the combined ON and OFF bipolar dysfunction, whereas cCSNB displays more myopia, likely relating to selective ON bipolar pathway dysfunction, with nyctalopia being similar in the 2 groups. Best-corrected visual acuity was preserved and stable over time, with more than half of the patients meeting driving license requirements in the UK, with longitudinal stability in retinal architecture on SD-OCT.

## Supplementary Data

Supplementary Methods

Supplementary Results
