# Supplementary material for: Congenital Stationary Night Blindness: Structure, Function and Genotype–Phenotype Correlations in a Cohort of 122 Patients
Source: Ophthalmol Retina. 2024 Sep;8(9):932–41. doi: 10.1016/j.oret.2024.03.017 (PMC11752838; doi:10.1016/j.oret.2024.03.017)
Supplement: Figure S1 [file mmc1.pdf]

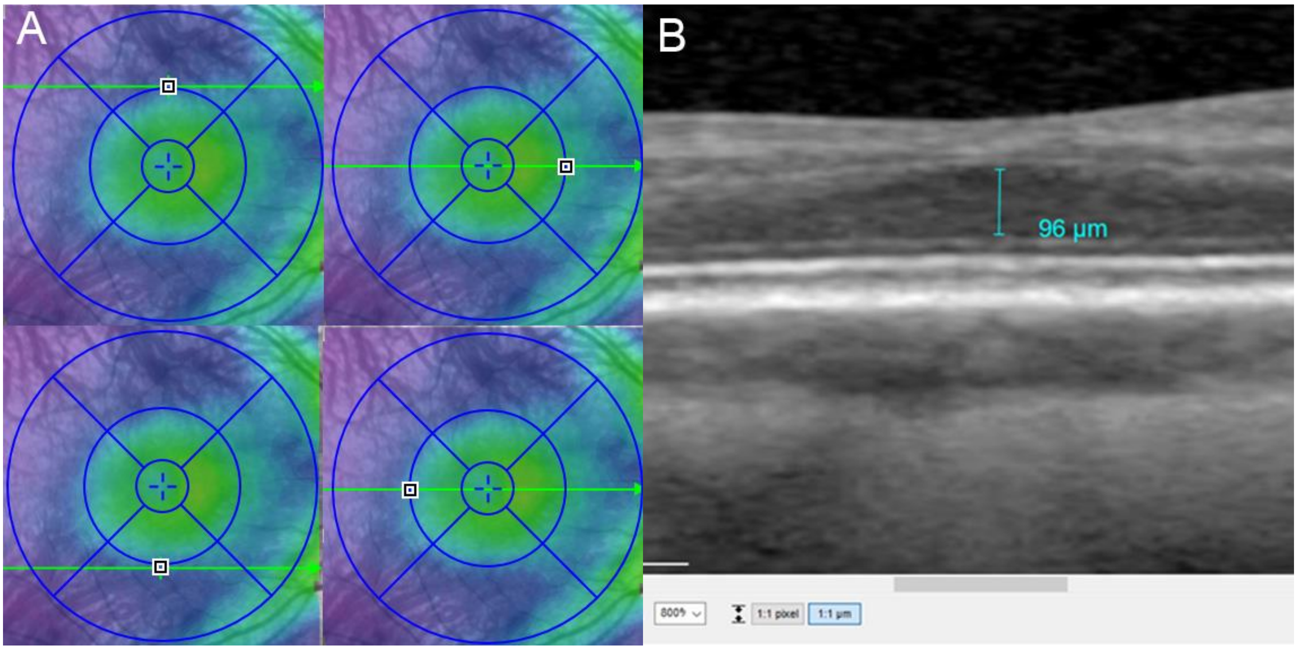

Supplementary Figure 1: OCT methodology.

Panel A: Ganglion cell layer plus inner plexiform layer (GCL+IPL) thickness was calculated by measuring the distance between the nerve fibre layer – ganglion cell layer interface and the inner plexiform-inner nuclear layer interface at four parafoveal points corresponding to the most superior/nasal/inferior/temporal points on the 3mm ETDRS circle. **Panel B:** Foveal outer nuclear layer thickness (ONL) was measured by setting the display ratio to 1:1µm and 800% magnification for more accurate measurement. The measurement was taken at the fovea from the border of the inner limiting membrane and the external limiting membrane. If there was concurrent foveal hypoplasia (persistence of the inner retinal layers over the fovea), as demonstrated in this patient, the measurement was taken from the outer plexiform – outer nuclear layer border to the external limiting membrane.
