# Supplementary material for: Congenital Stationary Night Blindness: Structure, Function and Genotype–Phenotype Correlations in a Cohort of 122 Patients
Source: Ophthalmol Retina. 2024 Sep;8(9):932–41. doi: 10.1016/j.oret.2024.03.017 (PMC11752838; doi:10.1016/j.oret.2024.03.017)
Supplement: Supplementary Methods [file mmc2.pdf]

Supplementary Methods - Genetics

All recruited patients were reassessed for their detected variants, (reference ID: *CACNA1F* – NM_005183.4, NP_005174.2; *CABP4* – NM_145200.5, NP_660201.1; *NYX* - NM_022567.3, NP_072089.2; *TRPM1* – NM_001252020.2, NP_001238953; *GRM6* – NM_000843.4, NP_000834.2; *GPR179* - NM_001004334.4, NP_001004334.3). Sequence variant nomenclature was obtained according to the guidelines of the Human Genome Variation Society (HGVS) by using Mutalyzer 2.0.[1] Classification of all detected variants was also performed based on the guidelines of the American College of Medical Genetics and Genomics (ACMG).[2,3] *In silico* molecular analyses was conducted. Minor allele frequency for the identified variants in the general population was assessed in the Genome Aggregation Database (gnomAD) datasets. General prediction scores were further calculated using MutationTaster, FATHMM, CADD, and REVEL. Functional prediction was performed employing SIFT, PROVEAN, and Polyphen 2. Human splicing finder 3.0 was applied for splicing defects prediction. Mammalian and vertebrate conservation were also investigated (PhyloP, PhastCons). The previously reported variants were surveyed with the HGMD database and ClinVar database (accessed on October 2023).

1. Lefter, M., Vis, J., Vermaat, M., den Dunnen, J., Taschner, P., and Laros, J. (2021). Mutalyzer 2: next generation HGVS nomenclature checker. Bioinformatics (Oxford, England) *37*. 10.1093/bioinformatics/btab051.
2. Richards, S., Aziz, N., Bale, S., Bick, D., Das, S., Gastier-Foster, J., Grody, W., Hegde, M., Lyon, E., Spector, E., et al. (2015). Standards and guidelines for the interpretation of sequence variants: a joint consensus recommendation of the American College of Medical Genetics and Genomics and the Association for Molecular Pathology. Genetics in medicine : official journal of the American College of Medical Genetics *17*. 10.1038/gim.2015.30.
3. Abou Tayoun, A., Pesaran, T., DiStefano, M., Oza, A., Rehm, H., Biesecker, L., and Harrison, S. (2018). Recommendations for interpreting the loss of function PVS1 ACMG/AMP variant criterion. Human mutation *39*. 10.1002/humu.23626.
