# Supplementary material for: Congenital Stationary Night Blindness: Structure, Function and Genotype–Phenotype Correlations in a Cohort of 122 Patients
Source: Ophthalmol Retina. 2024 Sep;8(9):932–41. doi: 10.1016/j.oret.2024.03.017 (PMC11752838; doi:10.1016/j.oret.2024.03.017)
Supplement: Supplementary Results [file mmc3.pdf]

Patients with Progressive Vision Loss

Only 4 patients lost 0.2 LogMAR visual acuity or more over the course of follow-up. Two of these patients (patient 86, *TRPM1* and patient 92, *TRPM1*) worsened by exactly 0.2 LogMAR(2 ETDRS lines) over 38 and 2 years follow up respectively. There was no indication of ocular co-morbidity on clinical examination or change in their OCT imaging. The third patient (patient 36, *CACNA1F*), worsened from 0.7 to 1.0 LogMAR, over 2 years of follow-up. Similarly, there was no observed change in the clinical examination or OCT imaging. The fourth patient (patient 49, *CACNA1F*), experienced a steady reduction in VA over 15 years of follow-up between age 15 to 30 from 0.5 to 1.5 LogMAR. Their SER was -12D at their last visit and this was the only refraction data available for this patient. There was no evidence in the clinical examination to explain the worsening VA. OCT imaging was also stable over the 9 year period that imaging was available.
