# Supplementary material for: Congenital Stationary Night Blindness: Structure, Function and Genotype–Phenotype Correlations in a Cohort of 122 Patients
Source: Ophthalmol Retina. 2024 Sep;8(9):932–41. doi: 10.1016/j.oret.2024.03.017 (PMC11752838; doi:10.1016/j.oret.2024.03.017)
Supplement: Table S1 [file mmc4.pdf]

Supplementary Table 1. CSNB patient demographics

Cross-sectional		First visit		Last visit		Follow-up	
Gene	Sex (Male, Female)	Mean, SD Years	Median, Years (Min, Max)	Mean, SD Years	Median, Years (Min, Max)	Mean, SD Years	Median, Years (Min, Max)
CACNA1F n=59	58 (100%), 0	10.48, 11.82	5.00 (1.0, 55.0)	18.60, 13.88	14.00 (2.0, 66.0)	8.13, 7.45	6.00 (0.0, 37.0)
NYX n=25	25 (100%), 0	8.74, 11.20	3.00 (0.7, 40.3)	15.76, 12.46	12.00 (3.0, 48.0)	7.02, 9.66	4.00 (0.0, 47.2)
TRPM1 n=21	9 (42.9%), 12 (57.1%)	9.45, 8.35	7.00 (0.7, 36.0)	17.71, 13.49	15.00 (4.0, 55.0)	8.26, 10.01	4.00 (0.0, 38.0)
GRM6 n=14	6 (42.9%), 8 (57.1%)	17.50, 18.38	6.00 (2.0, 49.0)	24.50, 16.43	18.50 (7.0, 53.0)	7.00, 6.23	5.50 (0.0, 22.0)
GPR179 n=2	1 (50.0%), 1 (50.0%)	1.13, 0.18	1.13 (1.0, 1.3)	12.50, 3.54	12.50 (10.0, 15.0)	11.38, 3.71	11.38 (8.8, 14.0)
CABP4 n=1	1 (100%), 0	3		7		4	
Total = 122	101 (82.8%), 21 (17.2%)	10.54, 12.17	5.00 (0.7, 55.0)	18.35, 13.78	14.00 (1.5, 66.0)	7.81, 8.16	5.00 (0.0, 47.2)

SD: Standard Deviation
