# Supplementary material for: Congenital Stationary Night Blindness: Structure, Function and Genotype–Phenotype Correlations in a Cohort of 122 Patients
Source: Ophthalmol Retina. 2024 Sep;8(9):932–41. doi: 10.1016/j.oret.2024.03.017 (PMC11752838; doi:10.1016/j.oret.2024.03.017)
Supplement: Table S2 [file mmc5.pdf]

Table 2: Longitudinal analysis demographics

Longitudinal visual acuity		First visit		Last visit		Follow-up	
Gene	Sex (Male, Female)	Mean, SD Years	Median, Years (Min, Max)	Mean, SD Years	Median, Years (Min, Max)	Mean, SD Years	Median, Years (Min, Max)
CACNA1F n=56	58 (100%), 0	10.78, 12.03	5.00 (1.0, 55.0)	19.14, 13.99	15.50 (2.0, 66.0)	8.37, 7.48	6.00 (1.0, 37.0)
NYX n=21	22 (100%), 0	7.03, 9.61	3.00 (0.7, 35.0)	15.38, 12.10	12.00 (3.0, 48.0)	8.35, 10.02	5.00 (2.0, 47.2)
TRPM1 n=20	9 (45%), 11 (55%)	9.63, 8.53	7.50 (0.7, 36.0)	18.30, 13.57	15.00 (4.0, 55.0)	8.68, 10.09	4.50 (1.0, 38.0)
GRM6 n=13	6 (50%), 6 (50%)	15.08, 16.65	5.00 (2.0, 47.0)	22.62, 15.45	18.00 (7.0, 53.0)	7.54, 6.13	6.00 (1.0, 22.0)
Total = 110	92 (83.6%), 18 (16.4%)	10.36, 11.75	5.00 (0.7, 55.0)	18.68, 13.71	14.50 (2.0, 66.0)	8.32, 8.28	5.10 (1.0, 47.2)
Longitudinal refractive error							
CACNA1F n=35	35 (100%), 0	7.70, 8.97	4.00 (1.0, 35.0)	16.88, 11.94	14.00 (2.0, 66.0)	9.16, 7.80	6.00 (1.0, 37.0)
NYX n=19	19 (100%), 0	6.56, 9.86	2.00 (0.7, 35.0)	14.95, 11.56	12.00 (3.0, 40.0)	8.39, 8.68	5.00 (2.0, 39.2)
TRPM1 n=19	9 (47.4%), 10 (52.6%)	8.76, 6.27	8.00 (0.7, 25.0)	16.95, 13.23	14.00 (4.0, 55.0)	8.18, 10.15	4.00 (1.0, 38.0)
GRM6 n=7	3 (42.9%), 4 (57.1%)	4.00, 1.63	4.00 (2.0, 7.0)	11.71, 3.50	12.00 (8.0, 16.0)	7.71, 4.07	6.00 (4.0, 14.0)
Total = 80	66 (82.5%), 14 (17.5%)	7.35, 8.22	4.00 (0.7, 35.0)	15.98, 11.62	13.00 (2.0, 66.0)	8.62, 7.26	5.10 (1.0, 39.2)
Longitudinal OCT							
CACNA1F n=26	26 (100%), 0	16.12, 12.50	12.21 (4.5, 56.0)	21.15, 12.32	16.91 (9.7, 58.0)	5.03, 3.40	4.63 (1.0, 14.0)
NYX n=7	7 (100%), 0	14.48, 5.19	15.00 (9.0, 24.6)	19.44, 5.67	19.79 (13.3, 29.0)	4.96, 1.90	4.49 (3.1, 9.0)
TRPM1 n=9	3 (33.3%), 6 (66.7%)	15.73, 8.40	13.35 (6.9, 36.0)	16.92, 4.95	17.00 (7.9, 25.0)	3.73, 1.83	3.45 (1.0, 6.0)
GRM6 n=7	3 (42.9%), 4 (57.1%)	20.49, 14.71	13.29 (6.0, 41.7)	24.03, 16.00	20.00 (7.0, 52.9)	3.54, 3.13	1.34 (1.0, 8.0)
GPR179 n=1	0, 1 (100%)	8		15		7	
CABP4 n=1	1 (100%), 0	16		17		1	
Total = 51	40 (78.4%), 11 (21%)	16.27, 11.08	13.29, (4.5, 56.0)	20.43, 11.01	17.00 (7.0, 58.0)	4.56, 2.97	4.3 (1.0, 14.0)
SD: Standard Deviation, OCT: Optical Coherence Tomography							
