# Supplementary material for: Congenital Stationary Night Blindness: Structure, Function and Genotype–Phenotype Correlations in a Cohort of 122 Patients
Source: Ophthalmol Retina. 2024 Sep;8(9):932–41. doi: 10.1016/j.oret.2024.03.017 (PMC11752838; doi:10.1016/j.oret.2024.03.017)
Supplement: Table S5 [file mmc6.pdf]

Supplementary Table 5. Mixed effects model of Best Corrected Visual Acuity (BCVA) by genotype and S-B subtype

Gene/Subtype	Predicted BCVA at birth (LogMAR, 95% CI)	Annual rate of change in BCVA (LogMAR, 95% CI)	Conditional Intraclass Correlation Coefficient (ICC)
CACNA1F n=56	0.56 (0.49, 0.63)***	-0.006 (-0.009, -0.003)***	
NYX n=21	0.65 (0.45, 0.84)	-0.012 (-0.021, -0.003)*	
TRPM1 n=20	0.47 (0.27, 1.01)	-0.004 (-0.012, 0.001)	
GRM6 n=13	0.42 (0.19, 0.66)	-0.009 (-0.018, 0.001)	0.567
Complete n=56	0.54 (0.48, 0.61)***	-0.009 (-0.012, -0.006)***	
Incomplete n=57	0.57 (0.41, 0.74)	-0.006 (-0.013, 0.001)	0.595
S-B: Schubert-Bornschein, *p*<0.05*, *p*<0.01**, *p*<0.001***			
