# Supplementary material for: Congenital Stationary Night Blindness: Structure, Function and Genotype–Phenotype Correlations in a Cohort of 122 Patients
Source: Ophthalmol Retina. 2024 Sep;8(9):932–41. doi: 10.1016/j.oret.2024.03.017 (PMC11752838; doi:10.1016/j.oret.2024.03.017)
Supplement: Table S6 [file mmc7.pdf]

Supplementary Table 6. Mixed effects model of Spherical Equivalent of Refraction (SER) by genotype and S-B subtype

Gene/Subtype	Predicted SER at birth (D, 95% CI)	Annual rate of change in SER (D, 95% CI)	Conditional Intraclass Correlation Coefficient (ICC)
CACNA1F n=35	-3.42 (-4.86, -1.98)***	-0.116 (-0.155, -0.077)***	
NYX n=19	-6.57 (-10.45, -2.68)*	-0.192 (-0.294, -0.089)*	
TRPM1 n=19	-6.19 (-10.09, -2.30)*	-0.163 (-0.260, -0.066)	
GRM6 n=7	-4.52 (-9.50, 0.46)	-0.198 (-0.187, 0.118)	0.738
Complete n=47	-5.92 (-7.20, -4.64)***	-0.162 (-0.192, -0.131)***	
Incomplete n=36	-3.24 (-6.45, -0.027)**	-0.117 (-0.196, -0.037)	0.778
TRPM1 males n=9	-5.68 (-12.70, 1.35)***	-0.098 (-0.246, 0.052)***	
TRPM1 females n=10	-7.21 (-10.02, -4.39)	-0.185 (-0.234, -0.135)	0.778
S-B: Schubert-Bornschein, *p*<0.05*, *p*<0.01**, *p*<0.001***			
