# Supplementary material for: Congenital Stationary Night Blindness: Structure, Function and Genotype–Phenotype Correlations in a Cohort of 122 Patients
Source: Ophthalmol Retina. 2024 Sep;8(9):932–41. doi: 10.1016/j.oret.2024.03.017 (PMC11752838; doi:10.1016/j.oret.2024.03.017)
Supplement: Table S7 [file mmc8.pdf]

Supplementary Table 7: Paired t-test results of OCT measures				
OCT measure	Mean (µm)	Std. Deviation	t (df=50)	Two sided *p*
First visit ONL thickness	93.53	13.66	1.535	0.131
Last visit ONL thickness	91.80	12.43		
First visit retinal thickness - central	260.58	20.11	-0.106	0.916
Last visit retinal thickness - central	260.65	20.89		
First visit retinal thickness - nasal	308.31	18.09	-0.730	0.469
Last visit retinal thickness - nasal	308.98	19.43		
First visit retinal thickness - inferior	302.73	17.84	1.517	0.135
Last visit retinal thickness - inferior	301.42	18.99		
First visit retinal thickness - temporal	295.10	16.70	-0.291	0.772
Last visit retinal thickness - temporal	295.40	17.50		
First visit retinal thickness - superior	304.77	18.51	-1.597	0.116
Last visit retinal thickness - superior	306.60	21.47		
First visit GCL+IPL thickness - nasal	76.96	10.69	0.393	0.696
Last visit GCL+IPL thickness - nasal	76.63	11.62		
First visit GCL+IPL thickness - inferior	67.63	11.13	-0.019	0.985
Last visit GCL+IPL thickness - inferior	67.65	12.57		
First visit GCL+IPL thickness - temporal	65.76	11.81	0.106	0.916
Last visit GCL+IPL thickness - temporal	65.67	10.45		
First visit GCL+IPL thickness - superior	67.16	10.79	-0.442	0.661
Last visit GCL+IPL thickness - superior	67.53	11.66		
OCT: Optical Coherence Tomography, Std: Standard, ONL: Outer Nuclear Layer, GCL+IPL: Ganglion Cell Layer + Inner Plexiform Layer				
