# Supplementary material for: Congenital Stationary Night Blindness: Structure, Function and Genotype–Phenotype Correlations in a Cohort of 122 Patients
Source: Ophthalmol Retina. 2024 Sep;8(9):932–41. doi: 10.1016/j.oret.2024.03.017 (PMC11752838; doi:10.1016/j.oret.2024.03.017)
Supplement: Table S8 [file mmc9.pdf]

Supplementary Table 8. Multivariate analysis of OCT measures between the S-B sub-types controlling for age and SER at the patient's last visit			
OCT measure	Mean difference (Incomplete - Complete)	Std. Error	Sig. p^b
Last visit ONL thickness	6.151	3.974	0.130
Last visit retinal thickness - central	13.316	6.911	0.061
Last visit retinal thickness - nasal	-2.901	6.158	0.640
Last visit retinal thickness - inferior	-3.532	5.497	0.524
Last visit retinal thickness - temporal	-6.750	5.310	0.211
Last visit retinal thickness - superior	-15.506*	6.791	0.028
Last visit GCL+IPL thickness - nasal	-7.628*	3.768	0.050
Last visit GCL+IPL thickness - inferior	-6.847	3.973	0.093
Last visit GCL+IPL thickness - temporal	-6.139	3.201	0.063
Last visit GCL+IPL thickness - superior	-4.654	3.979	0.249

OCT: Optical Coherence Tomography, S-B: Schubert-Bornschein, SER: Spherical Equivalent of Refraction, ONL: Outer Nuclear Layer, GCL+IPL: Ganglion Cell Layer + Inner Plexiform Layer, Std: Standard, Sig: Significance
b. Bonferroni adjustment for multiple comparisons
