# Supplementary material for: Congenital Stationary Night Blindness: Structure, Function and Genotype–Phenotype Correlations in a Cohort of 122 Patients
Source: Ophthalmol Retina. 2024 Sep;8(9):932–41. doi: 10.1016/j.oret.2024.03.017 (PMC11752838; doi:10.1016/j.oret.2024.03.017)
Supplement: Table S9 [file mmc10.pdf]

Supplementary Table 9. Summary of variant analysis												
	Number of variants		ACMG verdict assessment			Types of variants						
	Total	novel	Pathogenic	Likely pathogenic	VUS	Large deletion	Stop	Frameshift	Splicing	Missense	Inframe deletion/ insertion	Synonymous
CACNA1F	42	21	21	12	9	1	10	10	10	10	1	0
CABP4	1	1	0	1	0	0	0	1	0	0	0	0
NYX	19	11	0	10	9	0	1	3	0	12	2	1
TRPM1	27	14	5	9	13	1	1	2	7	16	0	0
GRM6	14	5	3	6	5	0	2	4	0	6	2	0
GPR179	2	1	1	0	1	0	0	1	0	1	0	0
Total	105	53	30	38	37	2	14	21	17	45	5	1
VUS: Variant of Unknown Significance												
