# Supplementary material for: Congenital Stationary Night Blindness: Structure, Function and Genotype–Phenotype Correlations in a Cohort of 122 Patients
Source: Ophthalmol Retina. 2024 Sep;8(9):932–41. doi: 10.1016/j.oret.2024.03.017 (PMC11752838; doi:10.1016/j.oret.2024.03.017)
Supplement: Table S10 [file mmc11.pdf]

Supplementary Table 10. Molecular genetic analysis

Variant No	Patient number	Gene	Transcript	Codon	Nucleotide change	Amino acid change	Sequence Ontology (Combined)	Effect (Combined)	ACMG Classification Verdict	Report name	Year	Novel
1	77	CACNA1F	NM_005183.4	Deletion of exon 2	Deletion of exon 2	NA	large deletion	LoF	Likely pathogenic	This study		Yes
2	52	CACNA1F	NM_005183.4	c.4059_4066delTCTTCAGGinsACACAGATAA	NM_005183.4:c.4059_4066delTCTTCAGGinsACACAGATAA	p.Leu1354HisfsTer3	Frameshift variant		Likely pathogenic	This study		Yes
3	54	CACNA1F	NM_005183.4	c.5416C>T	NM_005183.4:c.5416C>T	NP_005174.2:p.Gln1806Ter	Stop gained	LoF	Likely pathogenic	This study		Yes
4	17	CACNA1F	NM_005183.4	c.4723+1G>T	NM_005183.4:c.4723+1G>T	NA	Splice donor variant	LoF	Likely pathogenic	This study		Yes
5	13	CACNA1F	NM_005183.4	c.4645G>T	NM_005183.4:c.4645G>T	NP_005174.2:p.Glu1549Ter	Stop gained	LoF	Likely pathogenic	This study		Yes
6	57	CACNA1F	NM_005183.4	c.4577C>A	NM_005183.4:c.4577C>A	NP_005174.2:p.Thr1526Lys	Missense variant	Missense	VUS	Zeitz et al. Where are the missing gene defects in inherited retinal disorders? Intronic and synonymous variants contribute at least to 4% of CACNA1F-mediated inherited retinal disorders. Human Mutation.	2019	
7	20	CACNA1F	NM_005183.4	c.4424G>A	NM_005183.4:c.4424G>A	NP_005174.2:p.Gly1475Asp	Missense variant	Missense	VUS	This study		Yes
8	45	CACNA1F	NM_005183.4	c.4258T>G	NM_005183.4:c.4258T>G	NP_005174.2:p.Tyr1420Asp	Missense variant	Missense	VUS	This study		Yes
9	16, 43	CACNA1F	NM_005183.4	c.4084C>T	NM_005183.4:c.4084C>T	NP_005174.2:p.Arg1362Ter	Stop gained	LoF	pathogenic	Zeitz et al. Where are the missing gene defects in inherited retinal disorders? Intronic and synonymous variants contribute at least to 4% of CACNA1F-mediated inherited retinal disorders. Human Mutation.	2019	
10	12	CACNA1F	NM_005183.4	c.3887delG	NM_005183.4:c.3887delG	NP_005174.2:p.Arg1296Profs*41	Frameshift variant	LoF	Likely pathogenic	This study		Yes
11	11	CACNA1F	NM_005183.4	c.3633_3637delCTATGinsA	NM_005183.4:c.3633_3637delCTATGinsA	NP_005174.2:p.His1211GlnfsTer55	Frameshift variant	LoF	Likely pathogenic	This study		Yes
12	50	CACNA1F	NM_005183.4	c.3628C>T	NM_005183.4:c.3628C>T	NP_005174.2:p.Gln1210Ter	Stop gained	LoF	pathogenic	Carss KJ et al. Comprehensive Rare Variant Analysis via Whole-Genome Sequencing to Determine the Molecular Pathology of Inherited Retinal Disease. American journal of human genetic	2017	

13	41	CACNA1F	NM_005183.4	c.3525dupC	NM_005183.4:c.3525dupC	NP_005174.2:p.Lys1176Glnfs*18	Frameshift variant	LoF	Likely pathogenic	Zeitz et al. Where are the missing gene defects in inherited retinal disorders? Intronic and synonymous variants contribute at least to 4% of CACNA1F-mediated inherited retinal disorders. Human Mutation.	2019	
14	48	CACNA1F	NM_005183.4	c.3341_3342delCA	NM_005183.4:c.3341_3342delCA	NP_005174.2:p.Ser1114Cysfs*38	Frameshift variant	LoF	pathogenic	Carss KJ et al. Comprehensive Rare Variant Analysis via Whole-Genome Sequencing to Determine the Molecular Pathology of Inherited Retinal Disease. American journal of human genetic	2017	
15	3, 7, 18, 56	CACNA1F	NM_005183.4	c.3269+1G>A	NM_005183.4:c.3269+1G>A	NA	Splice donor variant	LoF	pathogenic	Hove MN et al. Clinical Characteristics, Mutation Spectrum, and Prevalence of Åland Eye Disease/Incomplete Congenital Stationary Night Blindness in Denmark. Investigative ophthalmology & visual science.	2016	
16	58	CACNA1F	NM_005183.4	c.3221C>T	NM_005183.4:c.3221C>T	NP_005174.2:p.Ser1074Leu	Missense variant	Missense	VUS	This study		Yes
17	40	CACNA1F	NM_005183.4	c.3213T>G	NM_005183.4:c.3213T>G	NP_005174.2:p.Asn1071Lys	Missense variant	Missense	VUS	This study		Yes
18	53	CACNA1F	NM_005183.4	c.3178C>T	NM_005183.4:c.3178C>T	NP_005174.2:p.Arg1060Trp	Missense variant	Missense	Likely pathogenic	Strom TM et al. An L-type calcium-channel gene mutated in incomplete X-linked congenital stationary night blindness. Nature genetics.	1998	
19	24	CACNA1F	NM_005183.4	c.3121_3122+2delAAGT	NM_005183.4:c.3121_3122+2delAAGT	NP_005174.2:p.?	Splice donor variant	LoF	Likely pathogenic	This study		Yes
20	27	CACNA1F	NM_005183.4	c.3069+2T>C	NM_005183.4:c.3069+2T>C	NA	Splice donor variant	LoF	Likely pathogenic	This study		Yes
21	9, 10, 29, 30, 36, 59	CACNA1F	NM_005183.4	c.3052G>A	NM_005183.4:c.3052G>A	NP_005174.2:p.Gly1018Arg	Missense variant	Missense	Likely pathogenic	Wutz K et al. Thirty distinct CACNA1F mutations in 33 families with incomplete type of XLCSNB and Cacna1f expression profiling in mouse retina. European journal of human genetics.	2002	
22	25	CACNA1F	NM_005183.4	c.3001G>T	NM_005183.4:c.3001G>T	NP_005174.2:p.Gly1001Ter	Stop gained	LoF	Likely pathogenic	This study		Yes
23	21	CACNA1F	NM_005183.4	c.2767-1G>T	NM_005183:c.2767-1G>T		Splice acceptor variant	LoF	Likely pathogenic	This study		Yes
24	37	CACNA1F	NM_005183.4	c.2932delC	NM_005183.4:c.2932delC	NP_005174.2:p.Arg978Glufs*27	Frameshift variant	LoF	Likely pathogenic	This study		Yes
25	35	CACNA1F	NM_005183.4	c.2822A>C	NM_005183.4:c.2822A>C	NP_005174.2:p.Asn941Thr	Missense variant	Missense	VUS	Zeitz C et al. Congenital stationary night blindness: an analysis and update of genotype-phenotype correlations and pathogenic mechanisms. Progress in retinal and eye research.	2015	
26	33	CACNA1F	NM_005183.4	c.2766+1G>A	NM_005183.4:c.2766+1G>A	NA	Splice donor variant	LoF	pathogenic	Carss KJ et al. Comprehensive Rare Variant Analysis via Whole-Genome Sequencing to Determine the Molecular Pathology of Inherited Retinal Disease. American journal of human genetic	2017	
27	31, 46	CACNA1F	NM_005183.4	c.2387_2388delAG	NM_005183.4:c.2387_2388delAG	NP_005174.2:p.Glu796Glyfs*51	Frameshift variant	LoF	Likely pathogenic	Zeitz et al. Where are the missing gene defects in inherited retinal disorders? Intronic and synonymous variants contribute at least to 4% of CACNA1F-mediated inherited retinal disorders. Human Mutation.	2019	
28	55	CACNA1F	NM_0	c.2173G	NM_005183.4:c.2	NP_0051	Missense	Missense	VUS	This study		Yes

			05183.4	>T	173G>T	74.2:p.Gly725Cys	variant					
29	22	CACNA1F	NM_005183.4	c.2071C>T	NM_005183.4:c.2071C>T	NP_005174.2:p.Arg691Ter	Stop gained	LoF	Pathogenic	Zeitz C et al. Congenital stationary night blindness: an analysis and update of genotype-phenotype correlations and pathogenic mechanisms. Progress in retinal and eye research.	2015	
30	34	CACNA1F	NM_005183.4	c.1910+1G>T	NM_005183.4:c.1910+1G>T	NA	Splice donor variant	LoF	Likely pathogenic	This study		Yes
31	4, 47	CACNA1F	NM_005183.4	c.1873C>T	NM_005183.4:c.1873C>T	NP_005174.2:p.Arg625Ter	Stop gained	LoF	pathogenic	Boycott KM et al. A summary of 20 CACNA1F mutations identified in 36 families with incomplete X-linked congenital stationary night blindness, and characterization of splice variants.Human genetics.	2001	
32	38	CACNA1F	NM_005183.4	c.1684+2T>C	NM_005183.4:c.1684+2T>C	NA	splice donor variant	LoF	Likely pathogenic	This study		Yes
33	32	CACNA1F	NM_005183.4	c.1538_1542delGAGCC	NM_005183.4:c.1538_1542delGAGCC	NP_005174.2:p.Arg513Glnfs*35	Frameshift variant	LoF	Likely pathogenic	Turro E et al. Whole-genome sequencing of patients with rare diseases in a national health system. Nature.	2020	
34	26	CACNA1F	NM_005183.4	c.1537C>T	NM_005183.4:c.1537C>T	NP_005174.2:p.Arg513Ter	Stop gained	LoF	pathogenic	Zeitz C et al. Congenital stationary night blindness: an analysis and update of genotype-phenotype correlations and pathogenic mechanisms. Progress in retinal and eye research.	2015	
35	8, 14	CACNA1F	NM_005183.4	c.1466_1496+7del	NM_005183.4:c.1466_1496+7del	NP_005174.2:p.?	splice donor variant	LoF	pathogenic	Turro E et al. Whole-genome sequencing of patients with rare diseases in a national health system. Nature.	2020	
36	28, 42, 44	CACNA1F	NM_005183.4	c.1218delC	NM_005183.4:c.1218delC	NP_005174.2:p.Trp407Glyfs*37	Frameshift variant	LoF	pathogenic	Stockner T et al. What can naturally occurring mutations tell us about Ca(v)1.x channel function? Biochim Biophys Acta.	2013	
37	2, 51	CACNA1F	NM_005183.4	c.1023delT	NM_005183.4:c.1023delT	NP_005174.2:p.Asp341Glufs*13	Frameshift variant	LoF	Likely pathogenic	This study		Yes
38	1, 5	CACNA1F	NM_005183.4	c.952_954delTTC	NM_005183.4:c.952_954delTTC	NP_005174.2:p.Phe318del	Inframe deletion	Missense	VUS	Zeitz et al. Where are the missing gene defects in inherited retinal disorders? Intronic and synonymous variants contribute at least to 4% of CACNA1F-mediated inherited retinal disorders. Human Mutation.	2019	
39	23	CACNA1F	NM_005183.4	c.868C>T	NM_005183.4:c.868C>T	NP_005174.2:p.Arg290Cys	Missense variant	Missense	VUS	Zeitz et al. Where are the missing gene defects in inherited retinal disorders? Intronic and synonymous variants contribute at least to 4% of CACNA1F-mediated inherited retinal disorders. Human Mutation.	2019	
40	19	CACNA1F	NM_005183.4	c.784C>T	NM_005183.4:c.784C>T	NP_005174.2:p.Arg262Ter	Stop gained	LoF	pathogenic	Carss KJ et al. Comprehensive Rare Variant Analysis via Whole-Genome Sequencing to Determine the Molecular Pathology of Inherited Retinal Disease. American journal of human genetic	2017	
41	39	CACNA1F	NM_005183.4	c.381+1G>A	NM_005183.4:c.381+1G>A	NA	splice donor variant	LoF	Likely pathogenic	This study		Yes
42	15	CACNA1F	NM_005183.4	c.244C>T	NM_005183.4:c.244C>T	NP_005174.2:p.Arg82Ter	Stop gained		pathogenic	Boycott KM et al.A summary of 20 CACNA1F mutations identified in 36 families with incomplete X-linked congenital stationary night blindness, and characterization of splice variants. Human genetics	2016	
43	122, 122	CABP4	NM_145200.5	c.61_62delCCinsA	NM_145200.5:c.61_62delCCinsA	NP_660201.1:p.Pro21Thrfs	Frameshift variant	LoF	Likely pathogenic	This study		Yes

						Ter6						
44	79	NYX	NM_022567.3	c.137T>G	NM_022567:c.137T>G	NP_072089.2:p.Val46Gly	Missense variant	Missense	VUS	Zeitz C et al. Congenital stationary night blindness: an analysis and update of genotype-phenotype correlations and pathogenic mechanisms. Progress in retinal and eye research.	2015	
45	66	NYX	NM_022567.3	c.293T>C	NM_022567.3:c.293T>C	NP_072089.2:p.Leu98Pro	Missense variant	Missense	Likely pathogenic	Simonsz HJ et al. Nightblindness-associated transient tonic downgaze (NATTD) in infant boys with chin-up head posture. Strabismus.	2009	
46	65	NYX	NM_022567.3	c.281delG	NM_022567:c.281delG	NP_072089.2:p.Arg94ProfsTer47	Frameshift variant	LoF	Likely pathogenic	This study		Yes
47	74	NYX	NM_022567.3	c.339_353del	NM_022567.2:c.339_353del	NP_072089.2: p.Glu114_Ala118del	Inframe deletion	Other	VUS			
48	76	NYX	NM_022567.3	c.411_419dup	NM_022567:c.411_419dup	NP_072089.2:p.Ala139_Cys140insTer	Stop gained	LoF	VUS	This study		Yes
49	69	NYX	NM_022567.3	c.425T>G	NM_022567:c.425T>G	NP_072089.2:p.Leu142Arg	Missense variant	Missense	Likely pathogenic	Hull S et al. Molecular and phenotypic investigation of a New Zealand cohort of childhood-onset retinal dystrophy. American journal of medical genetics. Part C, Seminars in medical genetics	2020	
50	61	NYX	NM_022567.3	c.523C>T	NM_022567:c.523C>T	NP_072089.2:p.Pro175Ser	Missense variant	Missense	VUS	This study		Yes
51	68	NYX	NM_022567.3	c.551T>C	NM_022567.3:c.551T>C	NP_072089.2:p.Leu184Pro	Missense variant	Missense	Likely pathogenic	Bech-Hansen NT et al. Mutations in NYX, encoding the leucine-rich proteoglycan nyctalopin, cause X-linked complete congenital stationary night blindness. Nature genetics.	2000	
52	72	NYX	NM_022567.3	c.647A>C	NM_022567:c.647A>C	NP_072089.2:p.Asn216Thr	Missense variant	Missense	VUS	This study		Yes
53	73	NYX	NM_022567.3	c.797T>A	NM_022567:c.797T>A	NP_072089.2:p.Leu266Gln	Missense variant	Missense	Likely pathogenic	Bech-Hansen NT et al. Mutations in NYX, encoding the leucine-rich proteoglycan nyctalopin, cause X-linked complete congenital stationary night blindness. Nature genetics.	2000	
54	70	NYX	NM_022567.3	c.854T>C	NM_022567:c.854T>C	NP_072089.2:p.Ile285Thr	Missense variant	Missense	Likely pathogenic	Bech-Hansen NT et al. Mutations in NYX, encoding the leucine-rich proteoglycan nyctalopin, cause X-linked complete congenital stationary night blindness. Nature genetics.	2000	
55	63	NYX	NM_022567.3	c.868_885dup	NM_022567:c.868_885dup	NP_072089.2:p.Glu290_Asn295dup	Inframe insertion	Inframe insertion	VUS	This study		Yes
56	80	NYX	NM_022567.3	c.951C>G	NM_022567.3:c.951C>G	NP_072089.2:p.Leu317=	Synonymous	Synonymous	VUS	This study		Yes
57	67	NYX	NM_022567.3	c.1028G>C	NM_022567:c.1028G>C	NP_072089.2:p.Arg343Thr	Missense variant	Missense	VUS	This study		Yes
58	83	NYX	NM_022567.3	c.1093C>T	NM_022567:c.1093C>T	NP_072089.2:p.Pro365Ser	Missense variant	Missense	VUS	This study		Yes
59	62	NYX	NM_022567.	c.1109G>T	NM_022567:c.1109G>T	NP_072089.2:p.Gl	Missense variant	Missense	Likely pathogenic	Pusch CM et al. The complete form of X-linked congenital stationary night blindness is caused by mutations in a gene	2000	

			3			y370Val				encoding a leucine-rich repeat protein. Nature genetics.		
60	82	NYX	NM_022567.3	c.1308dup	NM_022567.2:c.1308dup	NP_072089.2:p.Leu437AlafsTer56	Frameshift variant	LoF	Likely pathogenic	This study		Yes
61	62, 75, 77	NYX	NM_022567.3	c.647A>G	NM_022567.2:c.647A>G	NP_072089.2:p.Asn216Ser	Missense variant		Likely pathogenic	Zeitz C et al. Genotyping microarray for CSNB-associated genes. Investigative ophthalmology & visual science.	2009	Yes
62	71	NYX	NM_022567.3	c.803_807delins	NM_022567.2:c.803_807delins	NP_072089.2:p.Arg268LeufsTer81	Frameshift variant		Likely pathogenic	This study		Yes
63	115, 116	GRM6	NM_000843.4	c.2267G>A	NM_000843.4:c.2267G>A	NP_000834.2:p.Gly756Asp	Missense variant	Missense	Likely pathogenic	Sergouniotis PI et al. A phenotypic study of congenital stationary night blindness (CSNB) associated with mutations in the GRM6 gene. Acta ophthalmologica	2012	
64	112	GRM6	NM_000843.4	c.2155delC	NM_000843.4:c.2155delC	NP_000834.2:p.Arg719Glyfs*6	Frameshift variant	LoF	Likely pathogenic	This study		Yes
65	117	GRM6	NM_000843.4	c.2066delC	NM_000843.4:c.2066delC	NP_000834.2:p.Pro689Leufs*24	Frameshift variant	LoF	pathogenic	Dryja TP et al. Night blindness and abnormal cone electroretinogram ON responses in patients with mutations in the GRM6 gene encoding mGluR6. Proceedings of the National Academy of Sciences of the United States of America.	2005	
66	108, 108	GRM6	NM_000843.4	c.2030G>A	NM_000843.4:c.2030G>A	NP_000834.2:p.Arg677His	Missense variant	Missense	Likely pathogenic	Sergouniotis PI et al. A phenotypic study of congenital stationary night blindness (CSNB) associated with mutations in the GRM6 gene. Acta ophthalmologica	2012	
67	111	GRM6	NM_000843.4	c.1957C>T	NM_000843.4:c.1957C>T	NP_000834.2:p.Arg653Cys	Missense variant	Missense	VUS	This study		Yes
68	118, 118, 119, 119	GRM6	NM_000843.4	c.1934C>G	NM_000843.4:c.1934C>G	NP_000834.2:p.Pro645Arg	Missense variant	Missense	VUS	This study		Yes
69	109, 109	GRM6	NM_000843.4	c.1861C>T	NM_000843.4:c.1861C>T	NP_000834.2:p.Arg621Ter	Stop gained	LoF	pathogenic	Dryja TP et al. Night blindness and abnormal cone electroretinogram ON responses in patients with mutations in the GRM6 gene encoding mGluR6. Proceedings of the National Academy of Sciences of the United States of America.	2005	
70	114, 114	GRM6	NM_000843.4	c.1605delC	NM_000843.4:c.1605delC	NP_000834.2:p.Cys536Alafs*27	Frameshift variant	LoF	Likely pathogenic	This study		Yes
71	116	GRM6	NM_000843.4	c.1026C>A	NM_000843.4:c.1026C>A	NP_000834.2:p.Tyr342Ter	Stop gained	LoF	Likely pathogenic	This study		Yes
72	117	GRM6	NM_000843.4	c.824G>A	NM_000843.4:c.824G>A	NP_000834.2:p.Gly275Asp	Missense variant	Missense	VUS	Zeitz C et al. Genotyping microarray for CSNB-associated genes. Investigative ophthalmology & visual science	2009	
73	106, 106, 113, 113	GRM6	NM_000843.4	c.577delG	NM_000843.4:c.577delG	NP_000834.2:p.Val193Trpfs*16	Frameshift variant	LoF	pathogenic	Dryja TP et al. Night blindness and abnormal cone electroretinogram ON responses in patients with mutations in the GRM6 gene encoding mGluR6. Proceedings of the National Academy of Sciences of the United States of America.	2005	
74	110	GRM6	NM_000843.4	c.137C>T	NM_000843.4:c.137C>T	NP_000834.2:p.Pro46Leu	Missense variant	Missense	Likely pathogenic	Zeitz C et al. Night blindness-associated mutations in the ligand-binding, cysteine-rich, and intracellular domains of the metabotropic glutamate receptor 6 abolish protein trafficking. Human mutation.	2007	

75	110	GRM6	NM_000843.4	c.118_132delACGCTGGGCGGCCTG	NM_000843.4:c.118_132delACGCTGGGCGGCCTG	NP_000834.2:p.Thr40_Leu44del	Inframe deletion	Missense	VUS	Carss KJ et al. Comprehensive Rare Variant Analysis via Whole-Genome Sequencing to Determine the Molecular Pathology of Inherited Retinal Disease. American journal of human genetic	2017	
76	107, 107, 111	GRM6	NM_000843.4	c.58_72delTGGCTGGCGCAGGCG	NM_000843.4:c.58_72delTGGCTGGCGCAGGCG	NP_000834.2:p.Trp20_Ala24del	Inframe deletion	Missense	VUS	Taylor RL et al. Panel-Based Clinical Genetic Testing in 85 Children with Inherited Retinal Disease. Ophthalmology.	2017	
77	105, 105	TRPM1	NM_001252020.2	c.3622del	NM_001252020:c.3622del	NP_001238953:p.Glu1208ArgfsTer26	Frameshift variant	LoF	Likely pathogenic	This study		Yes
78	90, 91	TRPM1	NM_001252020.2	c.3538C>T	NM_001252020:c.3538C>T	NP_001238953:p.Arg1180Cys	Missense variant	Missense	VUS	This study		Yes
79	86	TRPM1	NM_001252020.2	c.3222T>A	NM_001252020:c.3222T>A	NP_001238953:p.Tyr1074Ter	Stop gained	LoF	pathogenic	Li et al. Recessive Mutations of the Gene TRPM1 Abrogate ON Bipolar Cell Function and Cause Complete Congenital Stationary Night Blindness in Humans. American journal of human genetic.	2009	
80	84, 84, 90, 90	TRPM1	NM_001252020.2	c.3206G>A	NM_001252020:c.3206G>A	NP_001238953:p.Cys1069Tyr	Missense variant	Missense	Likely pathogenic	Carss KJ et al. Comprehensive Rare Variant Analysis via Whole-Genome Sequencing to Determine the Molecular Pathology of Inherited Retinal Disease. American journal of human genetic	2017	
81	95	TRPM1	NM_001252020.2	c.3178+1G>A	NM_001252020:c.3178+1G>A	NA	splice donor variant	LoF	pathogenic	Audo I et al. Whole-exome sequencing identifies mutations in GPR179 leading to autosomal-recessive complete congenital stationary night blindness.Journal: American journal of human genetics.	2009	
82	90, 91	TRPM1	NM_001252020.2	c.3121A>T	NM_001252020:c.3121A>T	NP_001238953:p.Ile1041Phe	Missense variant	Missense	VUS	This study		Yes
83	100	TRPM1	NM_001252020.2	c.2939G>A	NM_001252020:c.2939G>A	NP_001238953:p.Arg980His	Missense variant	Missense	VUS	This study		Yes
84	100	TRPM1	NM_001252020.2	c.2912G>A	NM_001252020:c.2912G>A	NP_001238953:p.Cys971Tyr	Missense variant	Missense	VUS	This study		Yes
85	103	TRPM1	NM_001252020.2	c.2900G>A	NM_001252020:c.2900G>A	NP_001238953:p.Arg967Gln	Missense variant	Missense	VUS	Malaichamy S et al. Molecular profiling of complete congenital stationary night blindness: a pilot study on an Indian cohort. Molecular Vision.	2014	
86	87	TRPM1	NM_001252020.2	c.2765A>G	NM_001252020:c.2765A>G	NP_001238953:p.Glu922Gly	Missense variant	Missense	VUS	This study		Yes
87	102	TRPM1	NM_001252020.2	c.2245A>G	NM_001252020:c.2245A>G	NP_001238953:p.Thr749Ala	Missense variant	Missense	VUS	This study		Yes

88	96, 96	TRPM1	NM_001252020.2	c.2138+2T>C	NM_001252020:c.2138+2T>C	NA	splice donor variant	LoF	pathogenic	Li et al. Recessive Mutations of the Gene TRPM1 Abrogate ON Bipolar Cell Function and Cause Complete Congenital Stationary Night Blindness in Humans. American journal of human genetic.	2009	
89	99, 99	TRPM1	NM_001252020.2	c.2045T>C	NM_001252020:c.2045T>C	NP_001238953:p.Leu682Pro	Missense variant	Missense	VUS	This study		Yes
90	102	TRPM1	NM_001252020.2	c.1987C>T	NM_001252020:c.1987C>T	NP_001238953:p.Arg663Cys	Missense variant	Missense	Likely pathogenic	Nakamura M et al. TRPM1 mutations are associated with the complete form of congenital stationary night blindness. Molecular vision.	2010	
91	103	TRPM1	NM_001252020.2	c.1780C>T	NM_001252020:c.1780C>T	NP_001238953:p.Arg594Trp	Missense variant	Missense	VUS	This study		Yes
92	95	TRPM1	NM_001252020.2	c.1717G>A	NM_001252020:c.1717G>A	NP_001238953:p.Gly573Arg	Missense variant	Missense	Likely pathogenic	van Genderen MM et al. Mutations in TRPM1 are a common cause of complete congenital stationary night blindness. American journal of human genetics.	2009	
93	97, 97	TRPM1	NM_001252020.2	c.1140+3_1140+6del	NM_001252020:c.1140+3_1140+6del	NA	Splice region variant	Other	pathogenic	Nakamura M et al. TRPM1 mutations are associated with the complete form of congenital stationary night blindness. Molecular vision.	2010	
94	104	TRPM1	NM_001252020.2	c.1016G>A	NM_001252020:c.1016G>A	NP_001238953:p.Gly339Glu	Splice region variant	Other	Likely pathogenic	This study		Yes
95	89	TRPM1	NM_001252020.2	c.883G>A	NM_001252020:c.883G>A	NP_001238953:p.Gly295Arg	Missense variant	Missense	VUS	Carss KJ et al. Comprehensive Rare Variant Analysis via Whole-Genome Sequencing to Determine the Molecular Pathology of Inherited Retinal Disease. American journal of human genetic	2017	
96	104	TRPM1	NM_001252020.2	c.680T>A	NM_001252020:c.680T>A	NP_001238953:p.Val227Glu	Missense variant	Missense	VUS	This study		Yes
97	94, 94	TRPM1	NM_001252020.2	c.669+3_669+6del	NM_001252020:c.669+3_669+6del	NA	Splice region variant	Other	Likely pathogenic	Carss KJ et al. Comprehensive Rare Variant Analysis via Whole-Genome Sequencing to Determine the Molecular Pathology of Inherited Retinal Disease. American journal of human genetic	2017	
98	86	TRPM1	NM_001252020.2	c.533del	NM_001252020:c.533del	NP_001238953:p.Gly178ValfsTer10	Frameshift variant	LoF	pathogenic	Li et al. Recessive Mutations of the Gene TRPM1 Abrogate ON Bipolar Cell Function and Cause Complete Congenital Stationary Night Blindness in Humans. American journal of human genetic.	2009	
99	89	TRPM1	NM_001252020.2	c.431G>A	NM_001252020:c.431G>A	NP_001238953:p.Gly144Glu	Missense variant	Missense	VUS	Carss KJ et al. Comprehensive Rare Variant Analysis via Whole-Genome Sequencing to Determine the Molecular Pathology of Inherited Retinal Disease. American journal of human genetic	2017	
100	88, 88	TRPM1	NM_001252020.2	c.413T>C	NM_001252020:c.413T>C	NP_001238953:p.Leu138Pro	Missense variant	Missense	VUS	This study		Yes
101	85, 85	TRPM1	NM_001252020.2	c.135-3C>G	NM_001252020:c.135-3C>G	NA	Splice region variant	Other	Likely pathogenic	Nakamura M et al. TRPM1 mutations are associated with the complete form of congenital stationary night blindness. Molecular vision.	2010	
102	98, 100	TRPM1	NM_01252	c.54+1_54+18de	NM_001252020.2:c.54+1_54+18del	NA	Splice donor variant	LoF	Likely pathogenic	This study		Yes

			020.2	l								
103	98, 101	TRPM1	NM_0 01252 020.2	Deletion of exons 1-11	Deletion of exons 1-11	NA	large deletion	LoF	Likely pathogenic	This study		Yes
104	120, 120	GPR179	NM_0 01004 334.4	c.984del C	NM_001004334.4 :c.984delC	NP_0010 04334.3: p.Ser329 Leufs*4	Frameshift variant	LoF	pathogenic	Audo I et al. Whole-exome sequencing identifies mutations in GPR179 leading to autosomal-recessive complete congenital stationary night blindness. Journal: American journal of human genetics.	2012	
105	121, 121	GPR179	NM_0 01004 334.4	c.349G> T	NM_001004334.4 :c.349G>T	NP_0010 04334.3: p.Asp117 Tyr	Missense variant	Missense	VUS	This study		Yes
